# Mechanism and Therapies of Oxidative Stress-Mediated Cell Death in Ischemia Reperfusion Injury

**DOI:** 10.1155/2018/2910643

**Published:** 2018-06-24

**Authors:** Haobo Li, Zhengyuan Xia, Yanfang Chen, Dake Qi, Hong Zheng

**Affiliations:** ^1^The University of Hong Kong, Pok Fu Lam, Hong Kong; ^2^Harvard Medical School, Harvard University, Boston, MA, USA; ^3^Wright State University Boonshoft School of Medicine, Dayton, OH, USA; ^4^Memorial University of Newfoundland, St. John's, NL, Canada; ^5^The First Affiliated Hospital of Xinjiang Medical University, Xinjiang, China

Ischemia reperfusion-induced tissue injuries and organ failure represent the major causes of postoperative mortality and morbidity. Oxidative stress-mediated cell death plays a vital role in this pathology [1].

Induction of different types of cell death (e.g., apoptosis, necroptosis, and pyroptosis) triggered by reactive oxygen species (ROS) plays an important role in ischemia reperfusion injury (IRI) in multiple organs [2]. In this special issue, Z. Qiu et al. reported that, under hyperglycemic conditions, induction of nod-like receptor protein 3 (NLRP3) inflammasome-mediated pyroptotic cell death is critical in myocardial IRI, while inhibition of the inflammasome with specific inhibitors or ROS scavengers, N-acetylcysteine, reduced pyroptotic cell death and attenuated myocardial IRI. Moreover, oxidative stress-induced apoptotic and necroptotic cell deaths also play important roles in cardiac dysfunction as reported in this special issue by S. Peng et al. and N. Zeng et al, which showed that oxidative stress by increasing myocardial cell apoptosis and necroptosis leads to cardiac dysfunction in septic rats. Treatment with either PPAR-*γ* or brain-derived neurotrophic factor could reduce such types of cell death and attenuate cardiac dysfunction by reducing oxidative stress.

Reperfusion-induced oxidative stress is the major contributor in IRI. Thus, therapies that increase antioxidant capacity may protect organs against IRI [3]. Through its antioxidant capacity, the intravenous anesthetic propofol has been shown to alleviate myocardial IRI in patients undergoing cardiac surgery [4, 5] and in animals subjected to myocardial and intestinal IRI [6, 7]. In this special issue, F. Deng et al. reported that pretreatment with propofol to inhibit caveolae suppressed microvesicle release and attenuated cardiomyocyte hypoxia reoxygenation injury. Further, H.-J. Su et al. reported in this special issue that propofol conditioning confers antioxidative and cardioprotective effects against myocardial IRI through enhancing endogenous endocannabinoid release and the subsequent activation of CB2 receptor signaling. On the other hand, Z. Liu et al. reported that simvastatin pretreatment in donors may reduce hepatic oxidative stress through a fruppel-like factor 2-dependent mechanism which attenuated hepatic liver IRI and improved liver function recovery in rats that underwent liver transplantation. X.-T. Yan et al showed that treatment with PEP-1-heme oxgenase-1 fusion protein confers protection against septic shock-induced lung injury by reducing hepatic oxidative stress and inflammation, likely through suppression of toll-like receptor-4 and NF-*κ*B.

Aggravated inflammation, which has been shown to subsequently induce oxidative stress, has been proposed as a major cause of IRI and organ injury [8]. Attempts to attenuate organ injury by solely decreasing inflammation or increasing antioxidant capacity have achieved limited success [9, 10], indicating that multifaceted therapies combining anti-inflammatory and antioxidant approaches may be necessary for effective treatment. In this special issue, Q. Shan et al. reported that ingestion of 2,2′,4,4′-tetrabromodiphenyl ether (BDE-47), one of many persistent organic pollutants, leads to oxidative injury and cell death in the kidney by inducing the NLRP3 inflammasome. They further showed that application of troxerutin, a flavonoid with pharmacological antioxidant and anti-inflammatory activity, reduced BDE-47-indcued oxidative stress and cytotoxicity in the kidney through CXCR4/TXNIP/NLRP3 and Nrf2 signaling pathways. The findings of Q. Shan et al. may promote further in-depth studies regarding the interaction of inflammation and oxidative stress in the setting of IRI, which may facilitate our understanding of the pathophysiology of IRI and the development of new therapies for the disease.

We hope that the research articles presented in this special issue contribute to the understanding of current advancements and the mechanisms of oxidative stress-mediated cell death in ischemia reperfusion injury. It is also our hope to stimulate further efforts in the investigation of the pathology of ischemia reperfusion injury and the development of therapy for the disease.
